# Healthcare cost literacy in the united states: Cross-sectional estimates from a representative national sample

**DOI:** 10.1371/journal.pone.0354341

**Published:** 2026-08-19

**Authors:** Alexandra Guttentag, Smrithi Sukumar, Jeroen van Meijgaard, Khurram Nasir, Harlan Krumholz, Haider J. Warraich

**Affiliations:** 1 GoodRx Research, GoodRx, Santa Monica, California, United States of America; 2 Department of Medicine, Massachusetts General Hospital, Boston, Massachusetts, United States of America; 3 Houston Methodist Hospital, Houston, Texas, United States of America; 4 Yale University School of Medicine, New Haven, Connecticut, United States of America; 5 Yale Center for Outcomes Research and Evaluation, Yale-New Haven Hospital and Yale University, New Haven, Connecticut, United States of America; 6 Department of Medicine, Brigham and Women’s Hospital, Boston, Massachusetts, United States of America; Danube Private University, AUSTRIA

## Abstract

**Background:**

As healthcare costs and patient cost-sharing continue to rise in the United States, little is known about patients’ understanding of healthcare pricing, insurance-related expenses, and cost-planning strategies.

**Objective:**

To assess healthcare cost literacy (HCL) among U.S. adults and identify domains in which patients report the greatest and least confidence in understanding healthcare costs.

**Methods:**

A total of 2,308 American adults were surveyed. A cross-sectional design was utilized, incorporating a 12-item survey that examined key dimensions of healthcare cost literacy (HCL), such as healthcare cost knowledge, health insurance cost knowledge, and proactive cost planning behaviors.

**Results:**

Participants reported knowing most about pharmacy-level price differences for prescription drugs (77%) and how to contest a hospital bill (56%); they reported knowing least about hospital chargemasters (33%). When asked to describe various healthcare terms, participants found it easiest to describe “deductible” (mean 2.85 [SD 0.83]) and hardest to describe “health insurance formulary” (mean 2.10 [SD 0.96]). Regarding preemptive cost planning, participants had the most difficulty with knowing the cost of an emergency room visit ahead of time (mean 2.19 [SD 0.98]) and found it easiest to know the out-of-pocket cost of a prescription drug ahead of time (mean 2.60 [SD 0.87]). Participants with high-HCL, defined as those with>median HCL score of 2.42, were more likely to be older, have greater health literacy, and be more confident in managing their healthcare (all p < .001). They were more likely to have visited a doctor in the last year and were more knowledgeable about cancer screening guidelines (both p < 0.001).

**Conclusion:**

Levels of HCL are varied among the adult population, with wide knowledge gaps in understanding hospital bills and insurance-related costs. Efforts to bolster levels of HCL may help patients’ ability to anticipate, manage, and navigate healthcare expenses.

## Introduction

Over 23 million Americans owe medical debt [1]. And debt aside, Americans struggle with paying for healthcare; cost is a top-cited reason for nonadherence to medication treatment [2–4]. Understanding and planning for medical expenses is important, but can be difficult for patients; for example, a 2017 study found that 50% of people with healthcare expenses say their bills were more expensive than they anticipated [5]. Given that, in 2021, 32% of people could not pay an unexpected $400 bill [6], it is critical that individuals have the tools to limit these types of surprises. Despite nearly 90% of patients expressing desire for accurate information on healthcare services ahead of time, they may not know where to turn for this information [7].

Health literacy (HL) is defined by U.S. Health and Human Services as “the degree to which individuals have the capacity to obtain, process, and understand basic health information needed to make appropriate health decisions” [8]. Healthcare cost literacy (HCL) can be defined similarly, but with a specific addendum to cost: “the degree to which individuals have the capacity to obtain, process, and understand the costs associated with healthcare.” While similar in many aspects to health literacy, HCL remains understudied. Though surveys in the health literature and consumer research spaces have confirmed that medical cost uncertainty is a problem in the U.S, there is limited research on which subgroups within these insurance groups are most affected [9]. Further, it remains undetermined which components of the medical finance space Americans are most confident in managing, and in which spaces there are the largest understanding gaps. While health literacy is an oft-studied correlate of health behavior, the relationship between health literacy and HCL has not been explored.

Certain healthcare policies have been implemented to encourage increased price transparency to help patients anticipate and pay for care. Beginning in 2021, hospitals were required to provide an online list of their pricing for items and services [10]; this machine-readable menu is referred to as a hospital’s “chargemaster”. The Centers for Medicare and Medicaid Services also implemented the No Surprises Act in 2021, which provides protections from surprise billing for privately-insured Americans [11]. Despite these policies, the ability of patients to navigate this information is not tracked consistently. We set out to complement the existing literature on health literacy with an evaluation of knowledge related to health insurance, health care costs, and financial tools to mitigate cost, to provide a baseline measure of HCL.

Although research in the health literacy field has shown positive associations between health literacy and health decision making [12,13], little of the research discusses the financial components of healthcare management and planning. The purpose of this research was to assess general levels of HCL in the United States and its relation to health literacy; these findings can assist healthcare practitioners, insurers, and patient advocacy groups in developing materials and strategies for helping Americans where they need it most in the healthcare finance space.

In this paper, we describe current levels of HCL in the United States and how they relate to health literacy, with our null hypothesis that HCL is equivalent to HL. Through an evaluation of various components of HCL, we also make the case for the importance of further development and implementation of a validated scale for measuring individual HCL.

## Materials and Methods

This study was cross-sectional, based on a nationally-representative survey to assess how patients understand the costs of medical care. The survey was disseminated in two waves through the YouGov online survey platform in order to generate a larger sample size online survey panel platform (YouGov PLC, London, United Kingdom). The first wave was from 9/19/23–10/1/23 and the second wave was from 1/13/24–2/9/24. YouGov is a global public opinion and data company; the survey development methodology has been described in more detailed methodology via preprint [14]. The study was intended to be descriptive and exploratory in nature, and no formal a priori power calculation was performed; we collected an additional wave of data to increase sample size and improve precision of estimates. The first wave generated N = 1,500 responses, and the second wave generated N = 808 responses. The study was open to participants until a nationally representative sample of the U.S. adult population was achieved. Survey sampling and weighting procedures were designed to generate a sample representative of the U.S. adult population using the YouGov survey panel, which encompasses more than 9 million individuals [15,16]. Respondents were recruited from all major U.S. geographic regions and all 50 states. YouGov employed its standard sample matching methodology to generate a sample approximating the demographic composition of the U.S. adult population. Respondents were selected from the YouGov panel and matched to a target population frame based on demographic characteristics. Post-stratification weights were applied based on age, gender, race, education, and political affiliation.

All U.S.-based individuals were eligible for the survey, with the only inclusion criteria being that the respondent is an adult and provided consent. The survey contained 12 questions focused on HCL themes, including healthcare cost knowledge, health insurance cost knowledge, and preemptive cost planning [14]. We relied on the HLSQ-12 survey instrument as a framework for survey development and analysis [17]. Responses were based on a 4-point Likert scale and an HCL score was calculated as a participant’s mean score across all questions. Additional variables assessed included demographics and HL measured using the HLSQ-12 scale [17]. If a participant answered at least 75% of the HCL questions, any missing item scores were imputed using the mean score of their answered items. Participants who missed more than 25% of the HCL survey items were excluded from the analysis (N = 310).

We conducted univariate weighted analyses to describe responses to HCL survey items, and bivariate analyses (using chi-square tests or t-tests) to examine population-level differences between high and low HCL groups, defined by a median split of the HCL outcome variable. All analyses were performed using survey analysis software (srvyr package, version 1.2) in statistical software (R, version 4.4.1; R Foundation for Statistical Computing, Vienna, Austria) [18].

This study was reviewed by the independent Institutional Review Board (IRB) at Advarra, a leading IRB services company in North America. The study was determined to be exempt from further review in accordance with federal regulations (Pro00076816). Study participants provided written informed consent to the YouGov survey platform prior to beginning the survey, and assent included permission to publish anonymized results.

## Results

Of 2,308 respondents (median age 48, 51.3% female), the mean HL score across the sample was 2.98 (SD: 0.50); the mean HCL score across the sample was 2.43 (SD: 0.66).

Participants reported knowing most about pharmacy-level price differences for prescription drugs (77%) and how to contest a hospital bill (56%) (Table 1); they reported knowing least about hospital chargemasters (33%). When asked to describe various healthcare terms, participants found it easiest to describe “deductible” (mean 2.85 [SD 0.83]) and hardest to describe “health insurance formulary” (mean 2.10 [SD 0.96]). Regarding preemptive cost planning, participants had the most difficulty with knowing the cost of an emergency room visit ahead of time (mean 2.19 [SD 0.98]) and found it easiest to know the out-of-pocket cost of a prescription drug ahead of time (mean 2.60 [SD 0.87]). The 12-item HCL scale, while exploratory, demonstrated strong internal consistency (Cronbach’s α = 0.93); further details on exploratory factor analysis was previously published [14].

**Table 1:** Weighted Participant Responses to Questionnaire Assessing Healthcare Cost Literacy, Sample N = 2,308.

Theme	Question	% or Mean (SD)
**Cost Reduction Actions**	Have you ever asked your doctor for a cheaper alternative drug? (YES)	44.9%
	Have you ever asked your doctor about the cost of a medicine they were prescribing to you? (YES)	42.9%
**Healthcare Cost Knowledge**	Do you know that different pharmacies can charge different prices for same drug? (YES)	77.0%
	Do you know how to contest a hospital bill? (YES)	55.5%
	Do you know that the hospital chargemaster exists and can be requested/reviewed? (YES)	33.2%
**Health Insurance Cost Knowledge**	On a scale of very difficult (1) to very easy (4), how easy or difficult would you say it is to:	
	1) Describe what a health insurance deductible is?	2.85 (0.83)
	2) Describe the differences between a Health Savings Account (HSA) and Flexible Spending Account (FSA)?	2.25 (0.89)
	3) Describe the differences between coinsurance and copayment for insurance?	2.38 (0.91)
	4) Compare different insurance plan benefits to choose one that is best for me/ my family?	2.45 (0.88)
	5) Compare monthly cost and maximum deductible costs across different insurance plans during open enrollment time for health insurance?	2.31 (0.88)
	6) Describe what a health insurance formulary is?	2.10 (0.96)
	7) Describe the differences between a high deductible health plan (HDHP) and a traditional health plan?	2.47 (0.92)
	8) Describe what prior authorization is for insurance coverage?	2.82 (0.88)
**Preemptive Cost Planning**	9) Know how much a healthcare procedure will cost in advance?	2.21 (0.92)
	10) Know how much a primary care (non-urgent) visit will cost in advance?	2.55 (0.91)
	11) Know how much an emergency room or urgent care visit will cost in advance?	2.19 (0.98)
	12) Know what you will need to pay out of pocket at the pharmacy to fill a prescription drug?	2.60 (0.87)

Note: The 12 numbered questions were used to define healthcare cost literacy.

We used a median-split to classify participants as high-HCL versus low-HCL, informed by prior literature in the same or similar space [19,20]. As sensitivity analyses, we evaluated grop-level differences using quartile splits and using HCL as a continuous variable and the outcomes remained similar. Participants with high-HCL, defined as those with>median HCL score of 2.42, were more likely to be older, have greater HL, and be more confident in managing their healthcare (all p < .001) (Table 2). HCL and HL were moderately correlated (Pearson’s r = 0.60); those in the high-HCL group had an average HL score 0.45 points higher than those in the low-HCL group. Differences between the high-HCL and low-HCL groups included employment status (p < .001), insurance status (p < .001), self-rated health status (p < .01), and race/ethnicity (p < .01) (Table 2). Compared to those with low-HCL, those with high-HCL were more likely to have visited a doctor for an exam in the last year (74% vs 84%) and were more knowledgeable about cancer screening guidelines (61% vs 81%) (both p < 0.001).

**Table 2:** Weighted Sample Characteristics, Stratified by Median Level of Healthcare Cost Literacy (HCL).

	Level	<Median HCL	> Median HCL	P value
**Mean Age**		45.3	49.9	<0.001
**Gender**	Female	52.6%	49.5%	0.210
	Male	47.4%	50.5%	
**Race/Ethnicity**	Black	10.1%	14.0%	0.009
	Hispanic	16.2%	14.7%	
	White	63.5%	65.0%	
	Other	10.3%	6.3%	
**Highest Educational Attainment**	More than HS	64.9%	64.0%	0.703
	HS or Less	35.1%	36.0%	
**Employment Status**	Full-Time	40.9%	41.9%	<0.001
	Part Time	13.6%	12.0%	
	Retired	15.7%	26.4%	
	Unemployed	6.6%	5.2%	
	Other	23.1%	14.6%	
**Household Income**	Less than $30k	24.9%	21.8%	0.049
	$30-100k	52.4%	50.2%	
	$100k or more	22.7%	28.0%	
**Insurance Status**	Employer Sponsored	38.6%	34.4%	<0.001
	Exchange	6.6%	9.7%	
	Medicaid	17.0%	13.3%	
	Medicare	16.3%	27.6%	
	Uninsured	12.7%	8.1%	
	Other	8.8%	6.9%	
**Personal Health Evaluation**	Excellent-Good	73.6%	80.5%	0.001
	Fair-Poor	26.4%	19.5%	
**Mean Health Literacy Score**		2.73	3.18	<0.001
**Confidence In Managing Healthcare**	Confident	54.6%	92.3%	<0.001
	Not confident	45.4%	7.7%	
**Knowledge of when to start screening for breast or prostate cancer**	Yes	60.6%	81.4%	<0.001
	No	39.4%	18.6%	
**Visited a doctor for an annual visit in 2021**	Yes	73.7%	83.5%	<0.001
	No	26.3%	16.5%	
**Received COVID-19 Vaccine**	Yes	75.6%	79.4%	0.072
	No	24.4%	20.6%	

Results are presented as weighted percentages (%) or weighted means (standard deviation).

## Discussion

Our findings contradict the null hypothesis that healthcare cost literacy is equivalent to health literacy, as the two constructs were only moderately correlated (r = 0.60) and demonstrated distinct associations with demographic and healthcare utilization factors. Limited research has been done to assess current population-level understanding of the costs associated with medical care. An understanding of Americans’ levels of HCL will help policymakers and actors in the healthcare system develop targeted plans to educate consumers on financial planning and evaluation in the healthcare system. This study is the first in recent literature to survey US adults on their familiarity and confidence with navigating the financial components of the healthcare system.

Certain aspects of healthcare-related finances appear easier for patients to comprehend, and others are more challenging. The components that were most challenging were estimating costs ahead of time; namely for healthcare procedures and ER visits. This is concerning, given the reports that uncertainty around healthcare costs has led to individuals delaying or avoiding care when critical, even among those who are insured [21]. And while hospitals are required by law to publicly list their standard charges [22] (in a document called a “chargemaster”), per our findings only 33% of Americans know it exists. For patients that know about the chargemaster and wish to review chargemasters, the data is often messy and highly variable between hospitals in similar areas, making it difficult to compare costs and make informed clinical care decisions.[23,24]

Our findings showed that those with higher levels of HCL were an average of almost 5 years older than those with lower HCL. In the US, healthcare expenditures typically increase with age; adults in middle-age account for one-third of lifetime expenditures and seniors’ expenditures make up half of the total expenditure [25]. With age, individuals utilize the healthcare system more, and through incurring more charges may have a better understanding of medical finances. As medications and healthcare bills accumulate, they may also take more ownership into evaluating ways of saving, e.g., price shopping for medications.

Generally, we found that people with high levels of HL also had high levels of estimated HCL. In the lower HCL group, the mean HL score was 2.73 (scale of 1–4) versus 3.18 in the higher HCL group. Proven approaches for increasing HL, like personalized communication, focused educational materials, and task direction [26], might have similar effects on improving HCL as well.

The survey results also revealed that people with low levels of HL coupled with low levels of HCL had the greatest prevalence of poor health status. Independent of HL level, people with low levels of HCL were less likely to do a routine doctor’s visit, regardless of their health literacy categorization. To promote healthy wellbeing, it may be important to evaluate someone’s level of HCL both dependently and independently of their level of HL.

When stratifying by level of HCL (using a binary median split), we found that Black Americans made up 10% of the low HCL group and 14% of the high HCL group; this group also had higher raw scores of health literacy and HCL than other racial/ethnic groups. This is counter to published research showing that racial/ethnic minority groups have lower levels of health literacy [27]. There is a need for deeper qualitative and quantitative evidence bases to understand the interplay between race/ethnicity and HL and HCL.

There are limitations worth noting in our approach. Firstly, the survey data is cross-sectional, and therefore we were unable to confirm causal relationships between HL and the HCL questions assessed. Additionally, given that we compared factors individually between participants classified as high versus low HCL, we risk confounding among correlated factors; this may cause uncertainty with the estimates. Lastly, as with any survey, there may be response biases present. This survey was only offered to English-speaking respondents, effectively excluding a portion of the US population. Despite its limitations, there are important strengths to this work. Importantly, this work is novel; the research into cost literacy specifically associated with healthcare is very limited in nature. Secondly, the study is nationally representative, allowing us to draw conclusions and generate hypotheses that are generalizable to the United States.

Creating a validated measure of HCL for further research would serve the field well, as a step towards the development of and evaluation of interventions to improve HCL levels for those who need it most. Additionally, future research into HCL, using both qualitative and quantitative approaches on non-English speaking and immigrant groups could help provide important context for HCL challenges across the U.S. population.

## Supporting information

S1 Table Weighted Sample Characteristics, Wave 1 and Wave 2. (DOCX)

S1 Fig Histogram of Mean Healthcare Cost Literacy Scores. (DOCX)
