# Supplementary material for: Healthcare cost literacy in the united states: Cross-sectional estimates from a representative national sample
Source: PLoS One. 2026 Aug 19;21(8):e0354341. doi: 10.1371/journal.pone.0354341 (PMC13489418; doi:10.1371/journal.pone.0354341)
Supplement: Supplementary file 1 [file pone.0354341.s001.docx]

Supplementary Table 1: Weighted Sample Characteristics, Wave 1 and Wave 2

|  | **Level** | **Wave 1 (N=1,500)**  **%** | **Wave 2 (N=808)**  **%** |
| --- | --- | --- | --- |
| **Mean Age (SD)** |  | 47.3 (.51) | 49.1 (0.7) |
| **Gender** | Female | 51.9 | 50.2 |
|  | Male | 48.1 | 49.8 |
| **Race/Ethnicity** | Black | 13.7 | 14.0 |
|  | Hispanic | 15.4 | 14.7 |
|  | White | 61.7 | 65.0 |
|  | Other | 9.3 | 6.3 |
| **Highest Educational Attainment** | More than HS | 61.8 | 61.2 |
|  | HS or Less | 38.2 | 38.8 |
| **Employment Status** | Full-Time | 40.4 | 36.0 |
|  | Part Time | 13.6 | 12.5 |
|  | Retired | 20.1 | 25.1 |
|  | Unemployed | 7.0 | 7.5 |
|  | Other | 19 | 19 |
| **Household Income** | Less than $30k | 23.1 | 27.0 |
|  | $30-100k | 52.2 | 49.6 |
|  | $100k or more | 24.6 | 23.4 |
| **Insurance Status** | Employer Sponsored | 35.4 | 32.1 |
|  | Exchange | 7.6 | 8.3 |
|  | Medicaid | 15.0 | 17.6 |
|  | Medicare | 21.1 | 24.4 |
|  | Uninsured | 13 | 10.3 |
|  | Other | 8.0 | 7.3 |
| **Personal Health Evaluation** | Excellent-Good | 75.8 | 76.2 |
|  | Fair-Poor | 24.2 | 23.8 |
| **Mean Health Literacy Score (SD)** |  | 2.98 (0.5) | 2.98 (0.5) |
| **Confidence In Managing Healthcare** | Confident | 72.8 | 76.9 |
|  | Not confident | 27.2 | 23.1 |
| **Knowledge of when to start screening for breast or prostate cancer** | Yes | 70.2 | 72.5 |
|  | No | 29.8 | 27.5 |
| **Visited a doctor for an annual visit in 2021** | Yes | 75.3 | 79.6 |
|  | No | 24.7 | 20.4 |
| **Received COVID-19 Vaccine** | Yes | 75.3 | 76.8 |
|  | No | 24.7 | 23.2 |
