# Supplementary material for: Healthcare cost literacy in the united states: Cross-sectional estimates from a representative national sample
Source: PLoS One. 2026 Aug 19;21(8):e0354341. doi: 10.1371/journal.pone.0354341 (PMC13489418; doi:10.1371/journal.pone.0354341)
Supplement: Supplementary file 2 [file pone.0354341.s002.docx]

Supplementary Figure 1. Histogram of Mean Healthcare Cost Literacy Scores
